# A randomised controlled trial of a cognitive behavioural intervention for men who have hot flushes following prostate cancer treatment (MANCAN): trial protocol

**DOI:** 10.1186/1471-2407-12-230

**Published:** 2012-06-11

**Authors:** Omar Yousaf, Evgenia Stefanopoulou, Elizabeth A Grunfeld, Myra S Hunter

**Affiliations:** 1 Department of Psychology (at Guy's), Institute of Psychiatry, King's College London, 5 Floor Bermondsey Wing, Guy's Campus, London, SE1 9RT, UK; 2 School of Psychology, University of Birmingham, Birmingham, B15 2TT, UK

**Keywords:** Oncology, Cancer, Prostate, Self-help, Cognitive behaviour therapy, Protocol, RCT

## Abstract

**Background:**

This randomised controlled trial (RCT) aims to evaluate the effectiveness of a guided self-help cognitive behavioural intervention to alleviate problematic hot flushes (HF) and night sweats (NS) in men who are undergoing prostate cancer treatment. The trial and the self-help materials have been adapted from a previous RCT, which showed that a cognitive behavioural intervention reduced the self-reported problem-rating of hot flushes in women with menopausal symptoms, and in women undergoing breast cancer treatment. We hypothesize that guided self-help will be more effective than usual care in reducing HF/NS problem-rating at post treatment assessment.

**Methods/Design:**

Seventy men who are undergoing treatment for prostate cancer and who have been experiencing more than ten HF/NS weekly for over a month are recruited into the trial from urology clinics in London. They are randomly allocated to either a four-week self-help cognitive behavioural therapy (CBT) treatment or to their usual care (control group). The treatment includes information and discussion about hot flushes and night sweats in the context of prostate cancer, monitoring and modifying precipitants, relaxation and paced respiration, stress management, cognitive therapy for unhelpful thoughts and beliefs, managing sleep and night sweats, and advice on maintaining these changes.

Prior to randomisation, men attend a clinical interview, undergo 24-48-hour sternal skin conductance monitoring, and complete pre-treatment questionnaires (e.g., problem-rating and frequency of hot flushes and night sweats; quality of life; mood; hot flush beliefs and behaviours). Post-treatment measures (sternal skin conductance and the above questionnaires) are collected four-six weeks later, and again at a six-month follow-up.

**Discussion:**

MANCAN is the first randomised controlled trial of cognitive behavioural therapy for HF/NS for men that measures both self-reported and physiologically indexed symptoms. The results will inform future clinical practice by evaluating an evidence-based, non-medical treatment, which can be delivered by trained health professionals.

**Trial registration:**

UK Clinical Research Network UKCRN10904.

## Background

Prostate cancer is the most common cancer among men in the UK and the incidence rate has increased in the last 30 years, mainly due to improvements in the detection of the disease. While the five-year survival rate in the UK is good, prostate cancer survivors often face unwanted treatment side-effects, which are particularly troublesome following androgen deprivation therapy (ADT) [1]. Hot flushes and night sweats (HF/NS) are one of the main side-effects of ADT [2,3]. They are transitory sudden periods of heat and sweating lasting between two and ten minutes, generally on the neck, face and torso [4] The causal mechanism of HF/NS in men remains poorly understood, however, as for women, they are associated with changing hormone levels. A sudden change in levels of androgens is believed to alter the function of brain neurotransmitters (serotonin, noradrenalin and beta-endorphins), which in turn may lead to instability of the set point of the thermoregulatory centre in the hypothalamus. Intermittently, or in response to internal or external stimuli, the hypothalamus may down regulate body temperature by sweating and vasodilation [5].

HF/NS in men are under-researched compared to those experienced by menopausal women or women following breast cancer treatments. Up to 80% of men having ADT report HF/NS and these tend to be more frequent and severe than those experienced by women [6]. They can also be persistent; in one study over 40% of patients experienced HF/NS eight years post-treatment [7]. HF/NS are associated with distress and reduced quality of life – particularly affecting sleep and physical well-being [8]. The management of these symptoms presents a challenge to patients and clinicians alike. A recent systematic review of treatments for HF/NS in prostate cancer patients concluded that only a few treatments are available that are both effective and well tolerated, that more randomised controlled trials are needed, and that a priority should be the development of acceptable and effective treatments that are free from side-effects [5].

We have developed non-medical interventions for HF/NS [9,10] and a theoretical model of HF/NS [11]. This work identifies a range of factors that can moderate the intensity and experience of HF/NS, such as certain triggers (e.g., hot foods and drinks), stress, and cognitive/behavioural responses. There is evidence from laboratory studies with women that stressors increase the general level of HF reporting [12] and anxiety [13]. Also, unhelpful cognitions - that is negative thoughts associated with embarrassment, social anxiety, feeling out of control and unable to cope - are associated with more problematic HF/NS and sleep problems [14]. A psychological intervention, based on cognitive behaviour therapy (CBT) has been developed in the UK for women who have problematic HF/NS during the menopause transition [9] and for women following breast cancer treatment [10]. This has been found to be effective in reducing hot flush problem-rating (the extent to which they are problematic) in two recently published RCTs with breast cancer patients (MENOS1) [15] and well women (MENOS2) [16]. In MENOS2 both group CBT and guided self-help CBT were significantly more effective than no treatment, suggesting that a self-help format may be acceptable and beneficial. There is evidence that prostate cancer patients might prefer individualised informational support [17]; furthermore telephone support services are well received by men and guided self-help can be accessed by men living at a distance or who are housebound.

### Current study

This study aims to evaluate the effectiveness of guided self-help to alleviate HF/NS in men following treatment for prostate cancer. Guided self-help is compared to usual care (access to nurses and/or telephone support service) in a randomized controlled trial, using both physiological (sternal skin conductance [SSC]) and subjective measures of HF/NS and a 6 month follow-up. We hypothesise that guided self-help is more effective than usual care in reducing HF/NS problem-rating. Secondary analyses will also examine the effects of the treatment on HF/NS frequency, mood and quality of life (QOL). Mediating variables, including physiological HF/NS, beliefs and behaviours, are examined. A one year follow-up will be carried out by telephone off trial to estimate longer term outcomes. If effective, the treatment can be promoted by publication of the treatment manual and by training and supervising clinical nurse specialists in the application of the treatment.

The trial is funded by the Prostate Cancer Charity, and registered with the UK Clinical Research Network (UKCRN; Trial ID: 10904). NHS REC approval has been granted (South East London 2 REC, ref: 11/LO/1114) and local ethics and R&D approval has been obtained for recruitment of prostate cancer patients from all hospitals in the South East London Cancer Research Network (SELCRN).

## Methods/Design

### Study sample and recruitment

#### Inclusion criteria

Prostate cancer patients aged above 18, English-speaking, who have had more than 10 HF/NS weekly for at least one month will be included. Furthermore, screening takes place to ensure that patients consider their HF/NS problematic, and that their participation in the trial is motivated by their concerns about HF/NS and not any other problems, physical or psychological, that they are struggling with.

Patients are recruited into the treatment study by Prostate Cancer Nurse Specialists and Cancer Care Managers (Surviving Cancer Living Life telephone support service) at Guy's & St Thomas' NHS Foundation Trust, who provide a service to prostate cancer patients throughout the South East London Cancer Network. This catchment area includes a high proportion of African Caribbean men who are more likely to have prostate cancer. The men are contacted through medical staff at the clinics, and, after completing a telephone screening interview to assess inclusion criteria, they are provided with information about the study (verbal and written information about the two arms of the trial). Consent is then obtained for participation in the study (with a 2-week time interval). Following consent, they are invited for an interview where questionnaires are completed, and they are informed about their group allocation (guided self-help or usual care).

#### Procedure

The intervention: The guided self-help treatment is a 4-week intervention consisting of a booklet with information about prostate cancer and HF/NS, cognitive/behavioural exercises, and a CD with information, relaxation and paced breathing instructions. The approach is psycho-educational with individual treatment goals and an active focus upon cognitive and behavioural changes. The treatment includes:

· Information about causes of and factors affecting HF/NS.

· Monitoring and modifying precipitants, e.g. spicy food, alcohol.

· Relaxation and paced breathing, to reduce stress and apply at onset of HF/NS.

· Cognitive therapy for unhelpful thoughts and beliefs about HF/NS.

· Behavioural strategies to reduce stress and deal with HF/NS in social situations.

· Managing sleep and NS, drawing upon CBT for insomnia.

· Managing HF/NS and maintaining changes in the context of having prostate cancer.

At the initial assessment, which takes place in the Health Psychology department of King's College London at Guy's Hospital, the booklet is introduced and the patient is shown the paced breathing exercises. They are encouraged to discuss the treatment with their partner, family or friends. Two and four weeks later telephone interviews are held to discuss progress. Six weeks following assessment they are invited for a repeat assessment.

Usual care: The control arm receives standard care – they have access to their urologist and clinical nurse specialist, as well as cancer information and telephone support services. They thus have access to support and to existing written and verbal information about dealing with hormone related treatment side-effects. Once their participation in the trial ends, participants in the usual care group will be offered the intervention if they are interested.

Randomisation is carried out using the software Rand.exe version 6, which randomises participants to one of the two groups. Blocked stratification is used to ensure that the two groups do not differ on the potentially moderating variable of cancer type; depending on whether patients have localized or metastatic cancer (i.e., one that has spread to other organs), the intervention might be more or less effective. Patients with metastatic cancer may have lower levels of functioning and be less amenable to the intervention. Hence, stratifying for cancer type balances out any effects that might be due to this variable. Treatments undertaken during the follow-up period will be monitored in both arms of the trial.

**Figure 1:**
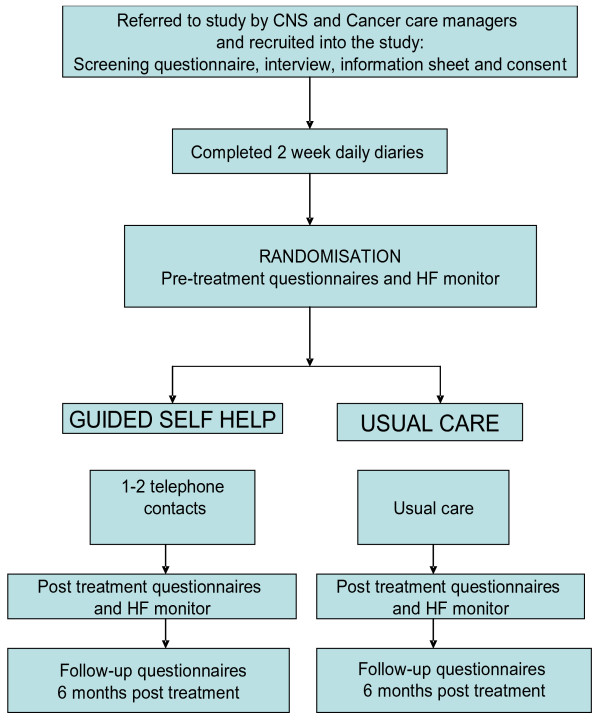
Study flowchart showing allocation to groups.

Blinding: During the screening and baseline assessments the clinical psychologist is blind to group allocation. After this, the researcher generates a randomised allocation using Rand.exe and sends this to the clinical psychologist who in the next meeting informs the patient of allocation and then either starts the intervention or informs patients that they will continue with their usual care. The post-intervention assessments are then carried out by the researcher who is blind to group allocation. Figure 1 shows the trial procedure.

### Measures

#### Socio-demographics

In the initial meeting the following data are registered: date of birth, height, weight, ethnicity, number of children, level of education, marital status, employment status, smoking, drinking, exercise participation, medication, and medical treatment history (including any treatments for HF/NS).

#### Primary outcome

The self-reported problem-rating of HF/NS (Hot Flush Rating Scale, or HFRS [18]) at post treatment will constitute the primary outcome; the score is the mean of 3 items (i.e., ‘*To what extent do you regard your flushes/sweats as a problem?*’, ‘*How distressed do you feel about your hot flushes?*’, and ‘*How much do your hot flushes interfere with your daily routine?*’) are rated on a 10-point Likert scale where a higher score indicates that the patient views his HF/NS as highly bothersome and interfering with life. A difference of 2 points or more is considered clinically relevant [9,10,19].The scale has good internal consistency in studies with women (Cronbach alpha=0.9) and test-retest reliability (r = 0.8). The problem-rating is chosen as the prime outcome measure because it, rather than frequency, is associated with help-seeking and quality of life, and in recent studies problem-rating has been recommended as the most appropriate patient reported outcome measure in clinical trials of HF/NS treatments [20,21]. Problem-rating and severity tend to be correlated and neither are strongly associated with frequency of HF/NS [21].

#### Secondary outcomes

The problem-rating of HF/NS described above will be measured again at the 6-month follow-up session as a secondary outcome.

Self-reported frequency of HF/NS is assessed by a subscale of the HFRS which measures the total number of HF/NS reported in the past week. This subjective HF/NS frequency measure was found to correlate (r = 0.9, p < 0.0001) with daily diary recordings of HF/NS in a previous study [18].

Quality of life (QOL) is assessed using both the 30-item EORTC QLQ-C30 [22] which is a standardised measure of QOL in cancer patients in general (including questions about physical and mental functioning and well-being), and the 25-item EORTC QLQ-PR25 [23] which is a prostate cancer-specific measure (which includes questions about urinary symptoms, sexual function, hormonal treatment-related symptoms etc.).

Mood is measured using the 14-item Hospital Anxiety and Depression Scale (HADS, [24]) which has two subscales for depressed mood and anxiety and was developed for use with medical samples. We expect that the intervention will have an effect on mood because hot flushes negatively affect patients’ mood and well-being.

Frequency of HF/NS, QOL and mood will all be measured at post-treatment and at the 6-month follow up assessment. Health economics questions, adapted from Beecham & Knapp [25]), including items on the number of consultations at primary and secondary care, absence from work, medical and non-medical treatments used across the duration of the trial, will be completed at the 6-month follow-up session.

#### Moderators

The 11-item Constructed Meaning Scale (CMS; [26]) measures whether, and/or how much, having a cancer diagnosis affects the way people feel about themselves, their relationships, and the future. We will examine whether men’s views about prostate cancer affect their response to the self-help treatment for HF/NS. This scale has previously been used in studies of people with cancer who vary in their tendency to perceive their cancer as a major threat [27], so there is a theoretical reason to predict that it might moderate the effectiveness of the intervention

The 4-item Emotional Control subscale of the Barriers to Help Seeking Scale (EC of the BHSS; [28]) measures a person’s willingness to express feelings and negative emotions. Including this measure enables us to explore whether beliefs about emotions are associated with outcome of the self-help treatment. Patients who are less willing to express emotions might be less amenable to the intervention which encourages an awareness of the relationships between cognitions, emotions and behaviours.

The 10-item Somatosensory Amplification Scale (SSAS; [29]) measures how sensitive individuals are to changes in their bodies. Including this as a moderator variable allows us to test whether heightened sensitivity affects the efficacy of the treatment. HF/NS may be exacerbated by an increased awareness of and attention to the body, so individuals who score highly on this scale might find it more difficult to follow some of the techniques of the treatment which aim to reduce attentional focus to HF/NS.

Finally, we employ the 10-item Revised Life Orientation Test (LOT-R; [30]) to examine whether trait optimism moderates the treatment outcome. This scale is used to assess how positive individuals’ outlook on life is, with a low score indicative of pessimism with regards to life events. Hence, individuals with low scores are not expected to benefit as much from the intervention as individuals with high scores on this scale.

#### Mediators

The Hot Flush Beliefs Scale [14] and The Hot Flush Behavior Scale [31] are used both before and after the treatment to check for any mediation effects. The scales, which were originally used for women with menopausal symptoms, have been modified for men (reduced to a combined total of 22 items). They measure types of beliefs and behaviours of individuals with HF/NS, some of which appear to be helpful and other less helpful [31]. Inclusion of these variables will help to clarify how the CBT intervention is working, i.e. whether specific beliefs and behaviours show change at post treatment and mediate improvement at follow-up.

The frequency of HF/NS is also measured by 24-48-hour ambulatory SSC monitoring (Bahr SSC monitor [Simplex Scientific; Middleton, WI, USA]) before and after treatment. The impact of the treatment upon subjective (frequency and problem-rating) and physiological measure of frequency will be an important secondary analysis and will help us to understand whether treatments are affecting the HF/NS threshold and/or symptom perception. We also record the self-reported frequency of relaxation practice, as well as treatment efficacy beliefs.

#### Statistical analysis

Primary efficacy analysis will be performed on the intent to treat population. The problem-rating of HF/NS at post treatment will be analysed using ANCOVA (i.e., analysis of covariance), adjusting for baseline problem-rating.

Secondary efficacy analyses: Secondary outcomes will be compared between groups as above. Possible mediators to response will also be investigated. The person performing the analyses will remain blind to the treatment grouping.

Safety analysis: The prevalence of specific adverse events is recorded and groups will be compared post-treatment.

Economic analysis: A descriptive analysis of economic data will be performed.

Statistical support may be sought from the Biostatistics Department, Institute of Psychiatry, King’s College London. Statisticians will not be informed of group allocation.

Power calculation: The sample size is calculated on the HF/NS Problem Rating Scale, estimating a mean of 5 (SD = 2.4) and a clinically relevant difference of 2 points [9]. A total sample size of 50, 25 in each group (allowing for 20% attrition) will have 90% power to detect a difference in mean HF/NS Problem-rating of 2 (a clinically significant change); based on results with women (no data on men exists) mean change of 2.87 and 0.77 in treatment and control groups respectively. However, we will recruit 70 (35 in each group) in order to power for secondary analyses, e.g. HF/NS frequency. We are recruiting from sites in the South East London Cancer Network and have badged the project with the NCRN. We estimate that 200 men receive ADT per year, that 35% (70) will meet inclusion criteria and that 16 months of recruitment should provide the numbers needed.

## Discussion

This study is a randomised controlled trial of cognitive behavioural therapy to treat hot flushes and/or night sweats experienced by prostate cancer patients currently undergoing treatment. The trial, due to its non-medical nature, might provide an acceptable alternative to medical treatments some of which have unwelcome side-effects [5]. The trial has been carefully designed to target key components that might reduce the impact of HF/NS [11]. We will be able to assess whether a four-week self-help CBT treatment results in significant improvements over usual care in terms of both self-reported and physiological measures of HF/NS.

We expect that guided self-help CBT will be more effective than usual care in reducing HF/NS problem-rating and secondary analyses will examine the effects of the treatment on HF/NS frequency, mood and quality of life (QOL). Moderators and mediating variables are examined including physiological HF/NS, beliefs and behaviours. A one year follow-up will be carried out by telephone off trial to estimate longer term outcomes. If effective, the treatment can be promoted by publication of the treatment manual and by training and supervising clinical nurse specialists in the application of the treatment.

### Methodological considerations

One of the strengths of the trial is that the intervention has been adapted from our previous work which proved successful in reducing the problem-rating and frequency of HF/NS in women [15,16]. There is no theoretical reason for differences in the mechanisms and triggers of HF/NS in men and women, so while the materials for the present intervention have been modified to suit men, the CBT components and focus on paced breathing and relaxation remain the same. The treatment is that it is standardised and replicable through the clearly specified manual, which step-by-step outlines how to deliver it according to the protocol. This will be important if and when the treatment is used in the future by trained health professionals.

Efforts have been made to include self-reported outcome measures that are directly related to the aspects that the intervention addresses. In addition, the inclusion of the physiological measure adds a more objective measurement of treatment efficacy. In terms of the study sample, it is demographically diverse, including men from both African Caribbean and White British backgrounds.

A limitation of the trial is that the usual care group, which serves as the control group, does not have any new treatment associated with it, which reduces its comparability to the self-help group. It may be the case that seeing a clinical psychologist and receiving two telephone calls, reduces the severity of HF/NS by, for example, reducing stress and uncertainty. Therefore, it would have been ideal with a control condition, e.g. a booklet with some non-CBT activities, to show that it is the self-help CBT, and not merely the support and attention, that reduces the problem-rating of HF/NS. However, this is an exploratory RCT and if CBT is found to be effective future research might include additional controls.

Another limitation of the trial is that of demand characteristics; patients might report improvements in the outcome measures simply to please the researchers. To reduce this problem, we ensure at the stage of the initial screening and first meeting that we do not present the trial as a stand-alone solution to their HF/NS but instead explain that the goal of the study is to assist patients in *managing* their symptoms, and not eliminating them. Also, we do not tell patients what the main outcome measure is.

## Conclusion

Prostate cancer survivors who are experiencing problematic HF/NS have few acceptable and effective treatment options that are free from side-effects. Cognitive behavioural therapy has shown significant reductions in HF/NS problem-rating and frequency in women [15,16]. This study is a randomised controlled trial of guided self-help CBT for HF/NS which includes both self-reported and physiological outcome measures. The trial tests for treatment effects on HF/NS, sleep, mood and health related quality of life. If effective, the CBT treatment for HF/NS might then be implemented widely by health professionals with relevant training and supervision.

## Competing interests

The authors declare that they have no competing interests.

## Authors’ contributions

OY is the trial coordinator of the study, and he prepared the first draft of this paper based on information from the study protocol. MSH is the principal investigator of the study, and she and EAG wrote the original protocol. ES is the clinical psychologist who runs the intervention, and she assisted in the process of preparing the final draft of this paper. All authors read and approved the final manuscript.

## Pre-publication history

The pre-publication history for this paper can be accessed here:

http://www.biomedcentral.com/1471-2407/12/230/prepub
